# Probabilistic assessment of human instability in urban areas exposed to flood events

**DOI:** 10.1038/s41598-025-25267-y

**Published:** 2025-11-24

**Authors:** Gabriele Bernardini, Francesco Lagona, Marco Mingione, Matteo Postacchini

**Affiliations:** 1 Università Politecnica delle Marche, DICEA, 60121 Ancona, Italy; 2 University of Roma Tre, DPS, 00145 Rome, Italy

**Keywords:** Civil engineering, Natural hazards

## Abstract

Floods in an urban environment are often associated with casualties among pedestrians who move to reach a safe area, due to body stability issues. Pedestrian instability is a random event that depends on the interaction between individual movement and floodwater. Randomness is however often overlooked by current methods, which assess instability risk by deterministic models and provide simplified thresholds that decision-makers then use in emergency planning. Here, instability risk is estimated by a logistic regression model where the toppling probability is a function of water depth and flow speed. The proposal depends on parameters that can be rigorously estimated by experimental data using standard statistical methods. It can be straightforwardly extended to allow for multiple instability mechanisms and subject-specific biometrical information. It includes previous proposals as particular cases, providing a general framework where different thresholds can be compared. It therefore provides a novel rigorous probabilistic integration of instability mechanisms into a unified logistic regression framework, calibrated with experimental data, and applied at urban scale.

## Introduction

Floods are one of the most relevant disasters globally, especially when they occur in urban areas^1–4^. On the one hand, urban areas may be placed in or near flood-prone areas (e.g., coasts, rivers, estuaries), evolving over time due to urban development and climate change effects^5–8^. On the other hand, urban areas can be characterized in terms of (i) physical vulnerability (that is, with reference to buildings and infrastructures), (ii) population exposure, and (iii) factors of social, economic and individual vulnerability^4,5,9–12^.

Flood risk assessment should accordingly encompass these factors, moving towards integrated multi-scale approaches, which can also improve the process of adaptation and mitigation of risk in urban areas^6^. Furthermore, flood risk management requires integrated tools to support decision-making and mitigation, addressing both physical impacts and socio-economic impacts^13–15^. In such context, modeling and analyzing flood hydrodynamics^16–20^ are basic tasks to evaluate flood-induced damage levels in buildings and infrastructures^21–24^, and to identify proper actions including structural and non-structural measures, such as physical interventions, land planning, the inclusion of nature-based solutions, insurance, awareness campaigns, etc.^4,12,25^. At the same time, these tasks are also useful to evaluate direct effects on the urban population, not only at a macro-scale level^4,5^, but also at the meso-scale level (i.e. neighborhood) and the micro-scale level (i.e. single building or part of open spaces, streets and squares)^11,26^.

Among the mentioned effects, those related to the interaction between individuals and floodwaters are noteworthy, especially under critical conditions that require evacuation^20,27–31^. These interactions are specifically relevant when individuals are (a) initially placed outdoors, in underground spaces or vehicles, or (b) cannot move up to look for a safe position (e.g. in single-storey buildings or public activities placed on the ground floor), or (c) are forced to move from their initial position after the beginning of the event^32–35^. Individuals are affected by surrounding/local floodwater conditions, i.e. water depth *D* [m] and speed *V* [m/s], which can slow pedestrians while moving, or even lead to loss of stability^20,29,30,36^. This second phenomenon is critical, as it can directly lead to potential injuries or casualties^3,37,38^. Its impact at the micro-scale level is also important because it relies on the specific local hydrodynamics induced by the floodwaters^27,29,30,35^. The inclusion of stability loss rules hence becomes fundamental not only in general risk assessment tools, but also in evacuation simulators^20,39–43^.

Broadly speaking, two main approaches have been proposed in the literature to describe pedestrian stability in floodwaters. The first relies on deterministic curves, which sharply divide stable from unstable conditions in the *D*–*V* plane^44–47^. Building on these, probabilistic models have been developed that draw on different input datasets and express hazard levels as a function of *D*–*V* pairs^47,48^. These models produce cumulative frequency distributions of instability events, often linked to damage functions, and have clear potential in risk assessment. In practice, they are sometimes used to generate flood maps that estimate the probability of pedestrian loss, thereby highlighting both safe zones and areas where preparedness and mitigation efforts should be prioritized^48,49^. Physical considerations on human stability in floodwaters are used to generate such curves, which lead to a balance between stabilizing (i.e. human weight and bottom friction) and destabilizing (i.e. buoyancy and drag force) actions. Two different mechanisms may occur: (a) toppling around a pivot, which can be either the foot toe (*Forward Toppling Instability*, hereafter FTI) or the heel (*Backward Toppling Instability*, hereafter BTI); (b) sliding or slipping. While the toppling mechanism is based on the moment balance around the pivot (i.e. either toe or heel), the sliding mechanism is described by the balance among all of the horizontal forces at play. However, such a horizontal balance is rather complex to physically recreate, especially due to the difficulty in correctly reproducing the resistive action induced by the friction between the pedestrian shoe and the ground. Being the sliding mechanism predominant on the toppling instability at high Froude numbers^45,50^ ($Fr=V/\sqrt{gD}$, with *g* being the gravity acceleration), its effect is taken into account through the velocity limitation in the $D-V$ plane^33^.

Assessment of these mechanisms has been performed using analytical models^45,46^, laboratory experiments^36,51^ and numerical models^50^. Specifically, Abt et al. ^51^ investigated the role of the critical $D-V$ product on the stability of human bodies, which is a function of body height and mass. This approach has been recalled by Jonkman and Penning-Rowsell ^44^, who estimated the probability distribution of the critical $D - V$ curve using two different datasets. Xia et al. ^45^ considered a statistical analysis to connect human body volume and mass, as well as to fit the experimental points found during dedicated laboratory tests. This physically based and experimentally calibrated method has been adjusted and updated by other research^52,53^, also to provide updated flood hazard assessment ratings^53^, and has been also implemented in different evacuation simulators coupling pedestrian movement and stability loss^39,41,43^. Furthermore, Milanesi et al. ^46^ started from the mentioned physical evaluation of the forces at play and then validated their model using available experimental data. In terms of thresholds for both *D* and *V*, Lind et al. ^54^ stated that, according to these “classical” approaches and analysis, the probability of toppling equals either zero or unity when, respectively, *D* is below 0.4*m* or above 1.2*m*, while many authors agree on a maximum *V* around $3\;m/s$^33,51^.

However, different main characteristics of the human body (e.g., weight, height, sex, mobility skills)^31,33,50,52,55,56^ seem fairly relevant for a suitable analysis of pedestrian stability in floodwaters and the relative definition of operational curves that can be employed in assessment tools and evacuation simulators^27,29,30,39^. Besides these issues related to individual features and vulnerability, additional uncertainties have been also observed during laboratory experiments^36^, demonstrating the possibility of different stability/instability results even under the same experimental input conditions. Research also demonstrated that differences in BTI and FTI stability thresholds exist^36,45^. As summarised by Table 1, existing approaches seem to be affected by some relevant limitations on the management of related uncertainties as well as on the input datasets about floodwaters direction. Deterministic rules do not directly include the randomness of body instability within the stability assessment curves on the $D - V$ plane. These uncertainty issues have motivated approaches based on three or more risk zones, based on the evaluation of people’s stability. in terms of “low”, “moderate”, “significant” and “extreme hazard”^33,45^. Similarly, Martinez et al. ^57^ analyzed some available datasets and categorized the following areas in their stability graphs: a “safety zone”, an “instability zone” and an “uncertainty zone”. The latter zone is between the first two areas and represents the potential risk of a pedestrian to become unstable^30^. Uncertainty management can be overcome by probabilistic methods^47,48^. Nevertheless, existing approaches generally seem to consider a single floodwater direction, or to not explicitly mention if probabilities are directly associated with FTI or BTI, or to move towards a separation between them in case BTI and FTI data are both used. On the contrary, the proposed model explicitly builds on experimental data on BTI and FTI and then merges them to create a unified probabilistic assessment model.

**Table 1:** Overview of main approaches on loss stability assessment under BTI and FTI conditions, in terms of basic parameters, managed uncertainties, floodwater direction and used datasets.

Approach	Basic flood parameters	Uncertainties	Floodwater directions and outcomes	Main references
Deterministic-multi-zone	D, V	Using different risk zones to manage uncertainties; no general explicit reference to uncertainties related to relationships between floodwater and pedestrian direction; providing different zones for different types of pedestrians (e.g., children)	Merging results from different datasets, or separating directions or not providing clear reference to input direction conditions	^33,45,57^
Probabilistic	D, V, Fr	Combining parameters in a general impact parameter (e.g. W) “to express the relative damage as a univariate function”^47^ (ranging continuously from 0 to 1, as the upper limit for stability of people); then calculating hazard degrees thanks to experimental data for different types of pedestrians (e.g., adults, children), according to isolines tracing specific values in the general impact parameter; finally providing relative damage curves (ranging from 0 to 1) to calculate expected loss, in function of the general impact parameter	Merging results from different datasets, or separating directions or not providing clear reference to input direction conditions	^47,48^
Our proposal	D, V	Defining continuous probability between 0 and 1 according to probabilistic approaches; further discussing discretization into multi-zone states depending on the probability range values; focused on typical adult conditions, but extendable to other pedestrian typologies	Using explicit experimental datasets on BTI and FTI by merging them in a unified model	

In this work, we account for the randomness of body instability by directly modeling the toppling probability as a logistic regression function of water depth and speed. In this sense, the proposed model explicitly and jointly builds on experimental data on BTI and FTI and, for the first time, merges them to create a unified probabilistic assessment model. Once calibrated on the available experimental data^36^, this method generates a continuous surface over the $D - V$ plane, which indicates the toppling probability *p* for each point of the plane. This allows, on one side, to specify the curve that splits the $D - V$ plane into the area where *p* is less than a given threshold $p^{\star }$, and the area where *p* is greater than $p^{\star }$, for any desired $p^{\star }$. On the other side, under this method, any curve on the $D - V$ plane receives a probabilistic interpretation and, as a result, previous proposals of toppling risk assessment^33,57,58^ can be viewed as particular cases and usefully compared.

In addition, our approach includes the typical advantages of a statistical model. First, it provides uncertainty measures that summarize the information content of the data. Models that assess instability risks must be calibrated on experimental data. Logistic regression methods allow to evaluate the information content of these data and the associated calibration quality, in contrast to deterministic methods. Second, and again differently from previous deterministic methods, logistic regression is capable to test the statistical significance of the effect of water depth and speed on the toppling probability and the statistical significance of differences between BTI and FTI conditions. Third, logistic regression predictions can be discretized, not only providing a quick tool for decision-makers and highlighting critical areas at the meso- and micro-scale during an evacuation, but also distinguishing risks under BTI and FTI conditions.

We use maximum likelihood methods to estimate the logistic regression function of interest. Maximum likelihood is the state-of-art tool to estimate probabilities of binary events (such as toppling versus non-toppling) under varying experimental conditions, and it requires single binary outcomes in a set of experiments carried out under varying floodwater conditions. In this sense, it is an easier and more flexible tool than methods that rely on frequencies of toppling events^44,47,48^.

The potential of our proposal is shown by applying the toppling risk assessment model with integrated BTI and FTI conditions to an urban area (a small neighborhood that includes some streets and squares), and comparing the results to those obtained by implementing existing approaches^33,57,58^.

## Methods

The methods are organized in four sections. We first introduce a binomial regression model with a logit link (or, briefly, a logistic regression model) that we use to specify the toppling probability as a function of water depth and speed. Second, experimental data for model calibration are resumed. Then, we illustrate the maximum likelihood methods that we use to estimate the model parameters from experimental data. Finally, we describe the hydrodynamics that we reproduced in a relevant urban area flood scenario, used here to obtain predictions of toppling probabilities in a urban domain.

### A logistic regression model of toppling risk

We model the toppling probability *p* as a logistic function of water speed (*V*) and water depth (*D*), indexed by four parameters $\beta _k, k=0,1,2,3$, say

1 $$\begin{aligned} p= \frac{\exp \left( \beta _0 +\beta _1 D+ \beta _2 V + \beta _3 DV\right) }{1+\exp \left( \beta _0 +\beta _1 D+ \beta _2 V + \beta _3 DV\right) }. \end{aligned}$$

Under (1), the probability *p* is always bounded between 0 and 1 for any value of the parameters $\beta$ and each point in the $D - V$ plane. For any threshold $p=p^{\star }$, (1) is a curve on the $D - V$ plane. It is the locus of the plane where the toppling probability is exactly $p^{\star }$ and splits the plane in a subset of conditions where the probability is less than $p^{\star }$ and a subset where the probability is greater than $p^{\star }$. In brief, for each fixed $p^{\star }$, equation (1) is a quantile curve that is often referred to as an instability curve, and we follow this terminology. The parameters $\beta$ modulate the log-odds of a toppling event, as it is apparent by writing model (1) in the following equivalent form:

2 $$\begin{aligned} \log \left( \frac{p}{1-p}\right) = \beta _0 +\beta _1 D+ \beta _2 V + \beta _3 DV. \end{aligned}$$

When $\beta _3=0$, the model reduces to a particular case when instability curves are linear. More generally, if $\beta _3 \ne 0$, the model allows for nonlinear instability curves. Model (1) can be estimated separately under BTI and FTI conditions. Our goal is however to test the differences between BTI and FTI conditions, therefore a joint model needs to be defined.

Specifically, let $\delta$ be a binary variable that indicates the flow direction, say $\delta =0$ for BTI and $\delta =1$ for FTI. Under this setting, the direction-specific toppling probability is modelled as

3 $$\begin{aligned} p_\delta = \frac{\exp \left[ (\beta _0+\beta _4 \delta ) + (\beta _1+\beta _5\delta ) D+ (\beta _2+\beta _6 \delta ) V+ (\beta _3 +\beta _7 \delta )D V\right] }{1+ \exp \left[ (\beta _0+\beta _4 \delta ) + (\beta _1+\beta _5\delta ) D+ (\beta _2+\beta _6 \delta ) V+ (\beta _3 +\beta _7 \delta ) DV\right] }, \qquad \delta =0,1, \end{aligned}$$

or equivalently,

4 $$\begin{aligned} \log \left( \frac{p_\delta }{1-p_\delta }\right) =&(\beta _0+\beta _4 \delta ) + (\beta _1+\beta _5\delta ) D+ (\beta _2+\beta _6 \delta ) V+ (\beta _3 +\beta _7 \delta )DV \nonumber \\ =&{\left\{ \begin{array}{ll} \beta _0 +\beta _1 D+ \beta _2 S+\beta _3 DV& \qquad \delta =0 \\ (\beta _0+\beta _4) + (\beta _1+\beta _5) D+ (\beta _2+\beta _6) V+ (\beta _3 +\beta _7)DV & \qquad \delta =1. \end{array}\right. } \end{aligned}$$

Under model (3), the parameters $\beta _0, \beta _1, \beta _2, \beta _3$ modulate the toppling log-odds under BTI, the parameters $\beta _0+\beta _4, \beta _1+\beta _5, \beta _2+\beta _6, \beta _3 +\beta _7$ drive the log-odds under FTI, and, finally, the parameters $\beta _4, \beta _5, \beta _6, \beta _7$ indicate the differences between FTI and BTI conditions. Compared to (1), (3) not only specifies a parametric family of instability curves on the $D - V$ plane that discriminate BTI and FTI conditions under which the toppling probability is less or greater than any given threshold $p^{\star }$. It also provides a general framework to test whether such curves are statistically different, on the basis of the available experimental data (see section on “Parameter estimation”).

### Experimental data

Our proposal depends on parameters that can be estimated by data that include occurrences of toppling events under controlled conditions of water flow speed, depth and direction, using standard statistical methods (see the following section on “Parameter estimation”). The data used here refer to the results of an experiment^36^ that adopted a quasi-natural scale mannequin representing a male individual with a height of 180 cm and a body mass of 80 kg, relying on existing anthropometric studies^55,59^. In particular, the mannequin was scaled to have a height of 112.5 cm and a total body mass of 19.57 kg, while floodwater conditions in experiments ranged from about 0.1 to 0.75 m for *D* and 0.3 to 2.4 m/s for *V*. This was done to represent real-world conditions ranging from about 0.16 to 1.20 m for *D* and from about 0.4 to 3.0 m/s for *V*, respectively. The mannequin included scaled feet, that allowed to hing it at the heel during BTI tests and at the toe during FTI tests. Tests were performed by dragging the mannequin through the floodwaters, for a battery of $D-V$ pairs. The occurrence of stable or unstable conditions was then recorded, and experiments were repeated, for each given pair and in the given BTI/FTI condition, at least five times. In fact, for specific $D-V$ pairs, it was noticed that experiments provided different toppling and non-toppling responses. For additional details about the experimental setting, we point the reader to the data source^36^.

### Parameters estimation

Maximum likelihood is the state-of-art method to estimate the parameters of a logistic regression model^60^. To illustrate, let *p* be the toppling probability. A toppling event can be modeled as a binary random variable *Y* that is equal to 1 if an object topples and 0 otherwise, with Bernoulli distribution $P(Y=y)=p(y)=p^{y}(1-p)^{1-y}$. If $y_1, \ldots , y_i, \ldots , y_n$ is a sequence of values taken by *Y* in *n* independent experiments, then *p* can be estimated as the most likely value, that is the maximum point of the log-likelihood function

$$\begin{aligned} \log L(p)= \log \left( \prod _{i=1}^{n}p^{y_i}(1-p)^{1-y_i}\right) . \end{aligned}$$

Under this setting, the maximum likelihood estimate (MLE) of *p* is given by the relative frequency of the toppling events observed among *n* experiments, say $\hat{p}=\sum _{i=1}^{n}y_i/n$. Its standard error is the inverse of the the square root of the curvature of the log-likelihood function at $\hat{p}$

$$\begin{aligned} \left[ \frac{\partial ^2 \log L(p)}{\partial p^2}\right] _{p=\hat{p}}^{-1/2} \end{aligned}$$

and indicates the precision of the MLE.

More generally, *Y* can be observed under different conditions (e.g. speed, depth and direction of the water flow), indicated by a row vector of *J* different experiment-specific conditions, say $\varvec{x}_i^\textsf{T}=(x_{i1}, \dots , x_{iJ})^\textsf{T}$. Under this setting, the toppling probability varies across experiments as a function of these conditions, say $p_i=p(\varvec{x}_i^\textsf{T})$. To examine the influence of experimental conditions on the toppling probability, a natural approach relies on assuming that probabilities are known up to a vector of regression parameters $\varvec{\beta }$ through a logistic regression function

$$\begin{aligned} p(\varvec{x}_i^\textsf{T}\varvec{\beta })=\frac{\exp (\varvec{x}_{i}^\textsf{T}\varvec{\beta }) }{1+\exp (\varvec{x}_{i}^\textsf{T}\varvec{\beta })}. \end{aligned}$$

Equations (1) and (3) are examples of the above specifications. The MLE of the regression parameters, $\hat{\varvec{\beta }}$ is the maximum point of the multivariate log-likelihood function

$$\begin{aligned} \log L(\varvec{\beta })=\log \left( \prod _{i=1}^{n}p(\varvec{x}_i^\textsf{T}\varvec{\beta })^{y_i}(1-p(\varvec{x}_i^\textsf{T}\varvec{\beta }))^{1-y_i}\right) , \end{aligned}$$

to be found by a Newton–Raphson algorithm. The standard errors of the regression parameters are obtained by computing the Hessian matrix $\varvec{H}_{\hat{\varvec{\beta }}}$ of the log-likelihood function $\log L(\varvec{\beta })$, evaluated at the MLE $\hat{\varvec{\beta }}$, and then taking the square root of the diagonal elements of the matrix $\varvec{H}_{\hat{\varvec{\beta }}}^{-1}$. Standard errors are computed to test the statistical significance of the estimated parameters. Once the MLEs $\hat{\varvec{\beta }}$ have been obtained, probability predictions $\hat{p}=\varvec{x}^\textsf{T}\hat{\varvec{\beta }}$ can be computed for any desired values of the design vector $\varvec{x}^\textsf{T}$. In particular, by plugging the MLEs into (1) and (3), we obtain predicted probabilities for each point of the $D - V$ plane or of more general domains that represent urban areas, like the one described in the next section.

### Application case study

**Fig. 1:**
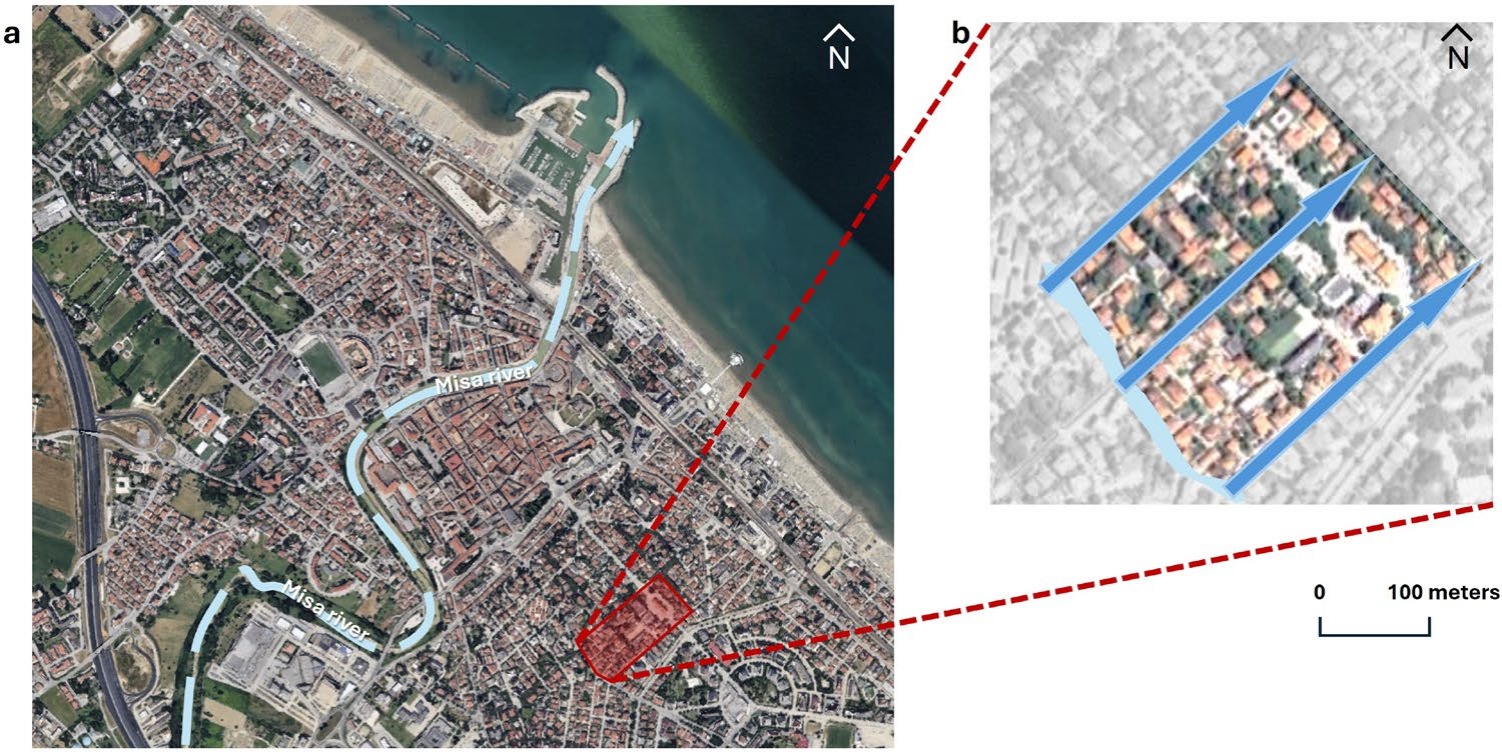
Plan view of the case-study area (red area) in the municipality of Senigallia (Italy), in panel (**a**), and detail view, including the main floodwater entrance location (light blue area) and main flow direction (blue thin arrows), in panel (**b**). Metric scale is applicable to panel b only. Base map from Immagini ©2024 Google, Immagini ©2024 Airbus, Maxar Technologies, Dati cartografici ©2024.

Figure 1 provides an overview of the study area where a flood was simulated to obtain predictions of toppling probabilities. This refers to an urban area within the center of Senigallia (Italy), a riverine town located along the Adriatic coast, which has suffered from many recent floods over the last 10 years. Senigallia is a coastal town, and a potential combination of multiple factors (e.g., rainfall, river flow, sea storm) may lead to extreme conditions for the urban population, implying possible activations of the evacuation process on foot. In particular, one of the most critical events in the selected urban area refers to the May 2014 flood, caused by a levee failure^29^. Floodwater spread along the urban streets and produced critical conditions within the selected urban area, entering the domain from the upstream part shown by the light blue area in Fig. 1 and then moving downstream along the main streets (big blue arrows).

The hydrodynamics were reproduced in previous research works referring to urban scenarios^29,40^ using a numerical solver based on the Nonlinear Shallow Water Equations (NSWE). Although the well-known limitations of depth-averaged shallow water models, such a solver was used for diverse purposes concerning urban, river, estuarine and coastal applications, resulting in an optimal choice for the simulation of shallow flows^29,61^. Here, it is exploited to obtain simulated $D-V$ maps in the area described in Fig. 1, at different times of the flood event.

## Results

In this section, the models are first fitted to the experimental outcomes^36^ separately for BTI and FTI (see Eq. (1)), and combining BTI and FTI conditions (see Eq. (3)). The proposed models account for the joint effect of water speed and depth, in addition to their marginal effects. Moreover, possible differences in these effects have been tested according to the water flow direction. Second, the fitted models are used to generate predicted probabilities in the selected area of Senigallia (Fig 1) to compare our results to the ones of previous reference works^33,57,58^.

### Logistic regression model to assess human body toppling instability in floodwaters

Table 2 reports parameters’ estimates (standard errors) resulting from the estimation of the proposed models. The first two columns display the estimates of model (1), using BTI and BTI data separately. The third column shows the estimates of the joint model (3). Stars ($*$) indicate that the estimate is significantly different from 0 at a $95\%$ confidence level and has therefore a non-negligible impact on evaluating the toppling risk.

As expected, while considering the models defined in Eq. (1), the marginal effects of floodwater *V* and *D* are positive, as shown by parameters $\beta _1$ and $\beta _2$, meaning that for a given value of speed/depth, the chances of toppling increase if depth/speed increases. While these estimates do not differ much between BTI and FTI conditions, a strong difference is observed for the interaction parameter $\beta _3$, which captures the curvature of instability conditions.

By estimating the the joint model (3), we are able to rigorously test (1) whether the marginal effects of *V* and *D* are not significantly different between BTI and FTI conditions and (2) whether the interaction effects are instead significantly different between BTI and FTI conditions. Results are displayed in the last column of Table 2 and indicate that only the interaction effect is significantly different between BTI and FTI conditions. In other words, the marginal effects of *V* and *D* on the toppling probability are the same regardless of how the person is standing, but their joint effect is reduced if the person is standing forward. This result could be essentially connected to the dynamics of FTI. In fact, as suggested in Postacchini et al.^36^, the FTI is influenced by “*a larger stabilizing moment due to a larger distance between the hinging location and the application point of the body weight*”.

The prediction reliability of the three models displayed in Table 2 is assessed by the traditional metrics of Table 3. Metrics are computed by comparing the number of most likely events that are predicted by each model with the number of observed events. The accuracy gives an overall measure of how good the model is at predicting either toppling or non-toppling events; the sensitivity, also known as true positive rate, measures how good the model is at predicting toppling events given that the toppling occurred; (iii) the specificity, also known as true negative rate, measures how good the model is at predicting non-toppling events given that the toppling did not occur. Table 3 highlights that BTI cases are uniformly easier to predict than FTI and that the model correctly classifies 83% of the data points overall, according to accuracy. Specificity values follow the same trend, by remarking that less than 20% of predictions might be incorrectly classified as toppling events (false positives). Additional results assessing the predictive accuracy of the model are reported in Section C of the Supplementary Information.

**Fig. 2:**
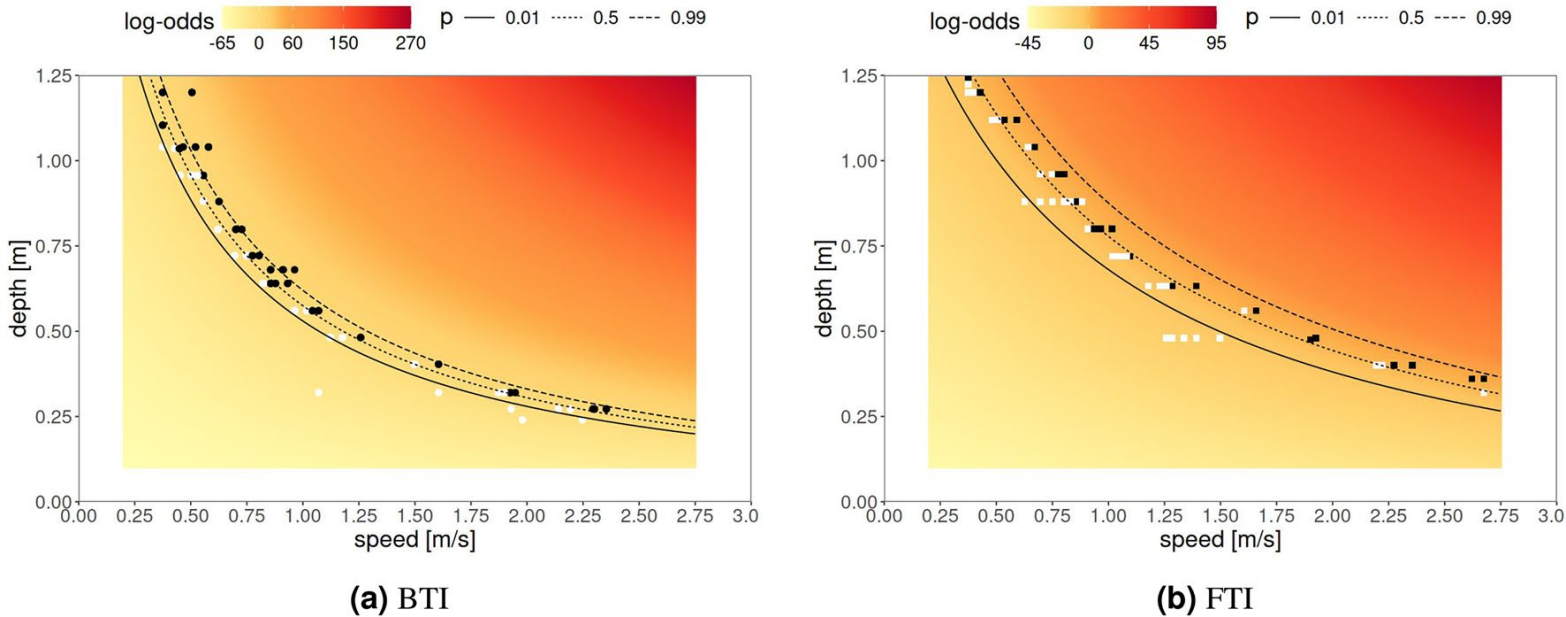
Toppling log-odds predicted across a grid of floodwater depth *D* and speed *V* values, fitting a logistic regression model to experimental data, for BTI (panel **a**) and FTI (panel **b**) conditions. Negative (positive) log-odds indicate a toppling probability less (greater) than 0.5. White and black dots respectively indicate observed non-toppling and toppling events. Curves indicate the loci of the points associated with given toppling probabilities *p*. Note that the color scale is different in the frames.

Figure 2 illustrates the toppling log-odds, as predicted by the model (3) across a grid of floodwater *V* and *D* values, under BTI (Fig. 2a) and FTI (Fig. 2b) conditions. The picture includes relevant instability curves obtained at $p*=0.01,0.5, 0.99$. The coloured area in the figures shows the (*V*–*D*) domain used for the design of the experimental tests, while the axes limits refer to stability limits commonly used in previous research works^33^, i.e. *V* ranging from 0 to 3 m/s and *D* ranging from 0 to 1.25 m. As shown by these instability curves, FTI-specific probabilities appear shifted upwards compared to their BTI counterpart, indicating that, for a given combination of *V* and *D*, toppling events seem to occur more often under BTI conditions than under FTI conditions.

**Table 2:** Maximum likelihood estimates and standard errors of the parameter of logistic regression models that predict toppling events, by analyzing BTI and FTI conditions separately (Columns 1 and 2) and jointly (Column 3). The $^*$ points to statistically significant estimates at the 95% confidence level (i.e., p-value < 0.05).

	BTI: Backward Toppling Instability	FTI: Forward Toppling Instability	BTI&FTI Toppling Instability
	Estimate (Std. Error)	Estimate (Std. Error)	Estimate (Std. Error)
$\beta _0$	$-68.184^*$ (12.712)	$-44.529^*$ (8.306)	$-68.184^*$ (12.712)
$\beta _1$	$4.223^*$ (1.474)	$4.628^*$ (1.343)	$4.223^*$ (1.474)
$\beta _2$	$26.693^*$ (5.507)	$23.008^*$ (4.666)	$26.693^*$ (5.508)
$\beta _3$	$84.582^*$ (15.778)	$28.264^*$ (5.357)	$84.582^*$ (15.778)
$\beta _4$			23.655 (15.185)
$\beta _5$			0.405 (1.994)
$\beta _6$			-3.686 (7.218)
$\beta _7$			$-56.318^*$ (16.663)

**Table 3:** Diagnostics of the logistic model.

	Accuracy	Sensitivity	Specificity
BTI	0.89	0.92	0.86
FTI	0.77	0.75	0.79
Overall	0.83	0.84	0.82

**Fig. 3:**
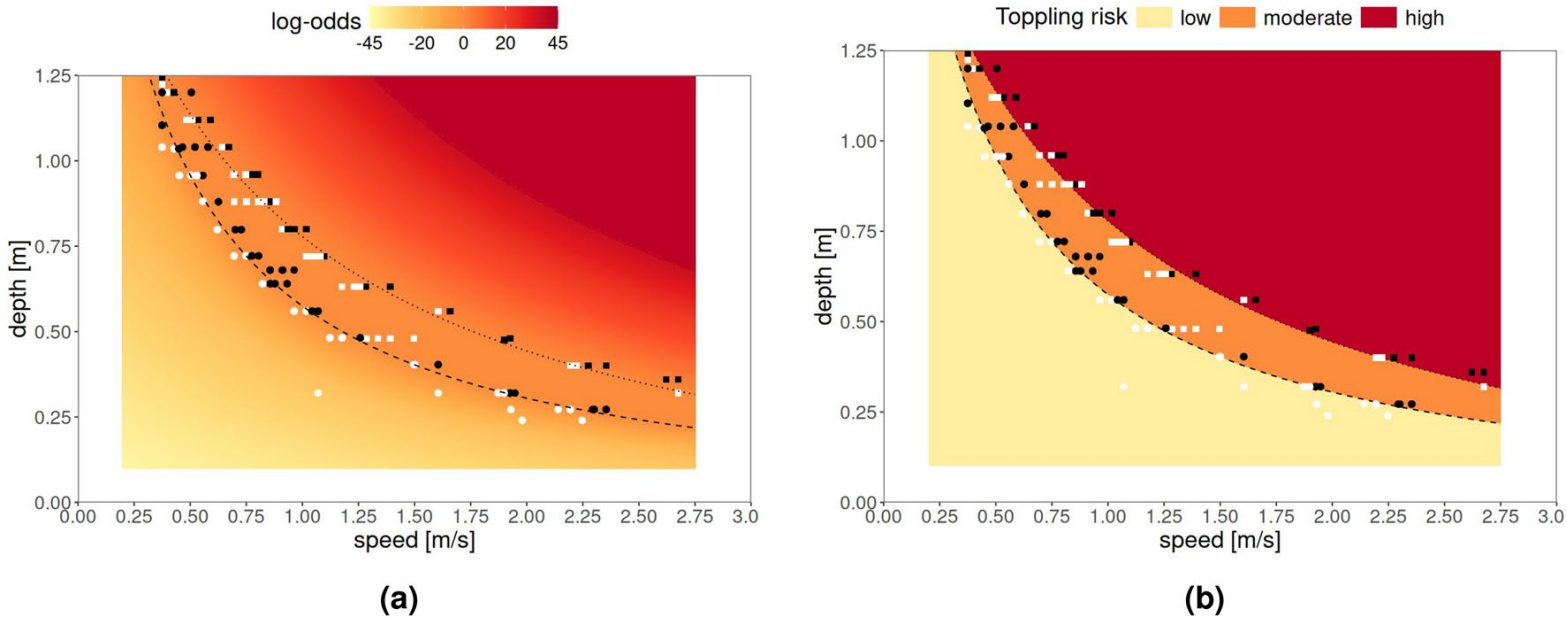
Toppling log-odds predicted across a grid of floodwater depth *D* and speed *V* values, fitting a logistic regression model to experimental data and averaged across BTI (circles) and FTI (squares) conditions, thus combining BTI and FTI conditions. Left panel (**a**): negative (positive) log-odds indicate a toppling probability less (greater) than 0.5. Right panel (**b**): predicted log-odds, clustered according to whether BTI and FTI log-odds are both negative (red), positive (yellow) or of discordant sign (orange), thus pursuing a three-zone approach for the sake of operational simplification and comparison with other existing discrete models. In both panels, curves indicate the loci of the points associated with a 0.5 probability under BTI (dashed) and FTI (dotted) conditions; white and black dots respectively indicate non-toppling and toppling events.

The predicted toppling probabilities displayed in Fig. 2 under BTI and FTI conditions can be combined in different ways. Figure 3 shows two useful approaches. Figure 3ashows the predicted toppling log-odds, $\log (\hat{p}/(1-\hat{p}))$, where $\hat{p}$ is the average between the predicted toppling probability under BTI and under FTI conditions, $\hat{p}=0.5 \hat{p}_\mathrm{{BTI}}+0.5\hat{p}_\mathrm{{FTI}}$, i.e the marginal toppling probability under the hypothesis that BTI and FTI are two equiprobable conditions. Figure 3binstead clusters toppling log-odds according to whether BTI and FTI log-odds share the same sign or not, indicating an uncertainty area where log-odds can be either positive or negative, depending on the relative direction of the water flow. Figure 3bcan be therefore seen as a *discretized* version of Fig. 3a, where:

- the red area shows the combinations of $D - V$ values for which “falling” is more likely than “not falling”, regardless of the position of the subject;
- the yellow area shows instead the combinations of $D - V$ values for which “not falling” is more likely than “falling”, regardless of the position of the person;
- the orange area represents an uncertainty area where “falling” is less likely than “not falling” under FTI conditions while “falling” is more likely than not falling under BTI conditions.

Figure 3btherefore offers a simple three-zone approach that simultaneously summarizes and integrate risks under BTI and FTI conditions. It not only provides an effective tool for decision-makers but also makes the approach comparable to other existing methods that rely on discrete conditions.

### Predicting toppling risk in an urban area

**Fig. 4:**
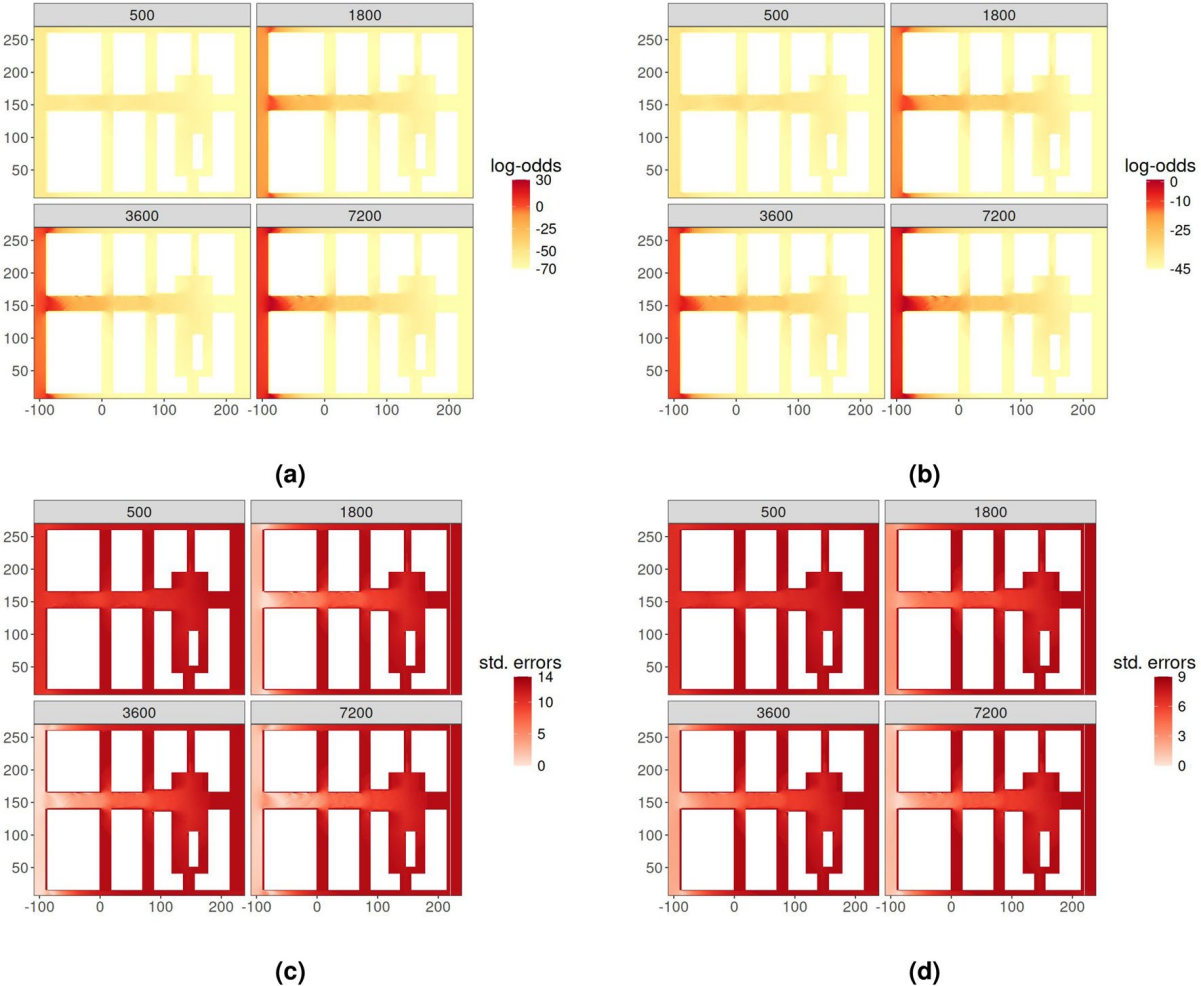
Log-odds (top) at different times (seconds) for BTI (left) and FTI (right) with associated standard errors (bottom). Note that the color scale is different in the frames.

The estimated models can be exploited to predict toppling probabilities in any $D - V$ domain of interest. Using the simulated hydrodynamics of a flood event in an urban area at four relevant time steps ($t = 500s, 1800s, 3600s, 7200s$; see Fig. A.1 of the Supplementary Information), we obtained four $D - V$ domains that we used for predicting the log-odds of a toppling event under BTI and FTI conditions during floodwater spreading (Fig. 4a,b ).

In both scenarios, the probability of toppling is nearly zero at $t = 500s$ in the whole area, since floodwater spreading is still limited, being $D-V$ pairs under the stability thresholds. Then, $D-V$ values increase at $t = 1800$ s, especially considering the left part of the urban area, which is close to the boundary where the river-overflow condition is assigned. It finally remains relatively constant at $t=3600$ s and $t=7200$ s. In general, the likelihood of toppling events is higher near the upstream boundary, particularly in the vicinity of the first two blocks and at their intersections. As the distance from the floodwater entrance location increases, the toppling probability declines, approaching zero on the opposite side (right boundary).

Figure 4c,d display the estimated standard errors of the predicted log-odds across the study area, respectively under BTI and FTI conditions. As expected, the uncertainty surrounding FTI toppling events is generally smaller than their BTI counterparts, reflecting the higher stability in FTI compared to BTI conditions. This result is also confirmed by the marginal distribution of the predicted log-odds (Fig A.2 of the Supplementary Information). In fact, as time evolves, the entropy of the toppling probabilities under FTI conditions is less than the entropy observed under BTI conditions and the FTI standard error distribution is shifted leftwards compared to its BTI counterpart.

**Fig. 5:**
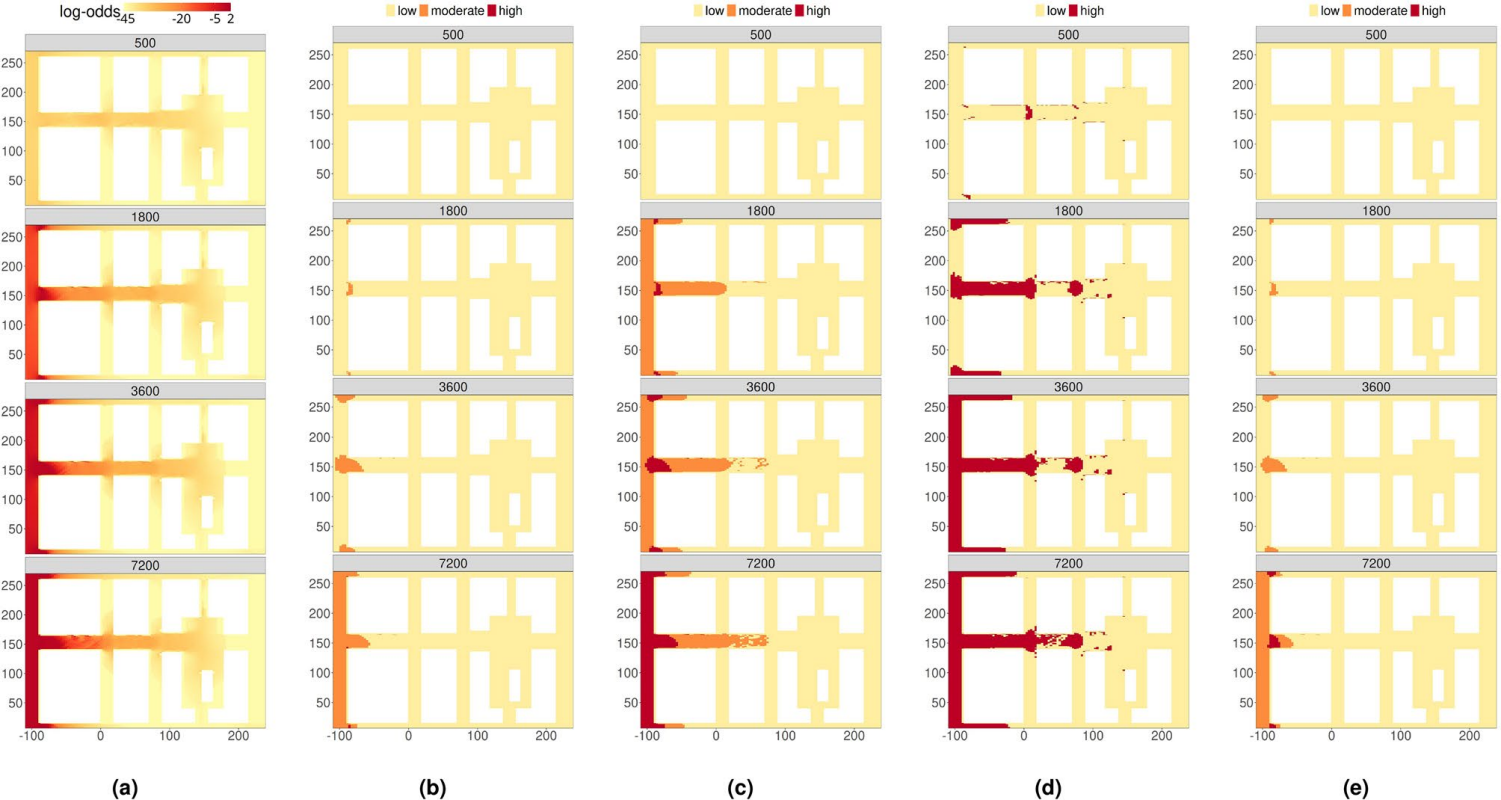
Comparison of different approaches for the evaluation of people instability at different times (seconds) during an urban flood: (**a**) proposed approach, (**b**) discretization of the proposed approach (**c**) Martínez-Gomariz et al.^57^ (**d**) Témez^58^ (**e**) Cox et al.^33^. The x and y coordinates of the grid are given in meters.

Our proposal provides a continuous surface of toppling risks with an accuracy that is not possible with traditional discrete methods^33,57,58^. However, when this surface is discretized according to whether BTI and FTI predicted log-odds share the same sign or not, we obtain results that are comparable with the previous literature. Specifically, Fig. 5 compares the continuous probability-based maps of combined FTI-BTI stability model of this work (Fig. 5a) and the related three-zone mapping (Fig. 5b), with the risk regions obtained by the three-zone approach (high/moderate/low-risk) by Martinez-Gomariz et al.^57^ (Fig. 5c), the two-zone (high/low risk) approach by Temez^58^ (Fig. 5d), and the four-zone (extreme/high/moderate/low-risk) approach by Cox et al.^33^ (Fig. 5e, which shows only three regions, as extreme risk is never reached). In particular, Fig. 5ashows results in terms of log-odds maps, being consistent with Fig. 3a, while Fig. 5bexpresses results according to the discretization of Fig. 3b, which is based on probability thresholds of 0.5 (or log-odds equal to 0). The threshold curves for each risk zone in the proposed model and in the previous literature methods are summarized in Fig B.1 of the Supplementary Information.

As expected, differences in toppling risk assessment are essentially due to the assumed threshold for risk zones in the compared models (i.e. see the reciprocal distance between the instability curves Fig B.1). Nevertheless, it is worth remarking that the proposed method identifies high-risk levels in very limited areas at/near the crossroads, especially for $t\ge 1800s$. A similar behavior is shared by the previous approaches with more than two risk regions^33,57^, which lead to high risk, especially at the crossroads. In this sense, the proposed model shows substantial similarities with the four-zone approach^33^, which represents a well-established and widely applied model.

At $t=7200s$, the whole system is under an almost steady flow condition^29^. Two main areas of interest can be noticed within the urban layout. Considering the flow entrance section (the first street on the left in each panel of Fig. 5), risk levels are higher than those in the other open spaces. Results from the current method are generally in line with those of the four-zone approach^33^, as in the previous *t* values, while three- and two-zones methods seem to overestimate risk levels^57,58^. Considering the main central street in the urban layout, risk levels decrease with the distance from the entrance section, as expected. The current method provides indeed risk values that are in line with those of the four-zone approach^33^. However, a smaller variation of risk conditions in the upstream part of this street, i.e. at the first crossroad, could be noticed in our method, while comparing $t=3600$ s and $t=7200$ s. This outcome is remarked by the very limited high-risk area pointed out in Fig. 5bat $t=7200$ s. Although the proposed discretization of the combined BTI-FTI model of this work seems to be less precautionary than the other approaches, results are still in line with the ones of the gold standard four-zone approach^33^ . In particular, as also remarked by Fig B.1 of the Supplementary Information, it can be noticed that this work only provides less conservative results within medium speed-depth ranges, i.e. for speed between 1.5 and 3.0 m/s and depth between 0.3 and 0.7 m. Hydrodynamic simulations in the central part of the first crossroad are characterized by such $D-V$ pairs. This is due, indeed, to assumed risk threshold curves as in Fig. 3b, and to the fact that $D-V$ pairs at the crossroad are included between the two curves associated with $p=0.5$ under each BTI and FTI condition. This area of Fig. 3bcorresponds to moderate risk and is characterized by a high uncertainty referring to the mutual direction between pedestrian motion and floodwater spreading. According to the experimental input data, FTI allows an increase in human body stability with respect to BTI. Including FTI in toppling assessment, thus, seems to balance risk levels including aspects related to “how” the pedestrian behaves in the urban layout. Such results could hence be more consistent with expected risks in view of pedestrian behaviours in evacuation conditions. In particular, in the central part of the upstream crossroads, pedestrians can activate specific safety behaviours^32,39^, i.e.: (1) limiting crossing the street in this area, preferring to move along building sides in view of attraction towards fixed obstacles; (2) adapting motion direction to floodwater flows, to decrease risk of stability loss in unprotected areas. Therefore, the individual arrangement in evacuation direction and trajectory can affect final effects on human body stability by reducing theoretical risk levels.

## Discussion and final remarks

This work provides different reliable models to accurately predict toppling instability for pedestrians moving in the same (FTI) and opposite (BTI) direction with respect to the incoming floodwater, as well as in the case of an uncertain motion direction. These models are obtained using experimental data from previous laboratory experiments^36^, and adopt a probabilistic approach in toppling events prediction. These continuous prediction outputs are also arranged into discrete risk zones for the merged BTI-FTI model, to provide a quick-to-apply tool for decision makers. The final results and the related methodology offer different advantages and implications, which have been showcased in an urban area prone to flood, selected as a relevant case study application. In particular, prediction outcomes are compared to other existing methods, demonstrating the capabilities of the new proposed approach, thus encouraging future works to overcome current limitations.

### Including pedestrian and floodwater direction in toppling risk assessment model

From a modeling perspective, the probabilistic approach adopted here is based on logistic regression. Although simple, this choice is relatively new compared with consolidated methods to which our results are benchmarked^33,57,58^, as well as with other probabilistic approaches available in the literature^47,48^. To the best of the authors’ knowledge, this is the first application of a logistic model that jointly incorporates BTI and FTI conditions within a single framework. This innovation allows predictions of toppling events that account for the actual direction of pedestrian movement, rather than relying on unidirectional or unspecified datasets. The model produces continuous probability estimates of stability loss (see Fig. 3a), which could be further organized into a multi-zone representation (see Fig. 3b), and it can be readily embedded in evacuation models where pedestrian trajectories and floodwater flows are combined through behavioural-hydrodynamic simulations.

The proposed method however presents some differences with the well-established multi-zone method by Cox et al.^33^, regarded here as the reference standard. In particular, our logistic formulation produces more conservative risk estimates in scenarios characterized by relatively low velocities (below about 1 m/s) and high depths (above 0.8 m). This is mainly due by accounting for the distinction between FTI and BTI conditions and is consistent with the expectation that, under quasi-still flows, differences between FTI and BTI are limited, especially considering the dominance of the moment criterion^44^. Conversely, in medium *D*–*V* ranges—roughly 0.3–0.7 m in depth and 1.5–3.0 m/s in velocity—the model yields less conservative estimates. These conditions correspond to the greatest separation between FTI and BTI curves at $p=0.5$, suggesting that individuals may adjust their movement direction to mitigate hydrodynamic effects, thereby lowering effective risk levels. At the same time, the relatively small distance among the curves confirms both the robustness of the three-zone discretization derived from our probabilistic formulation and the reliability of Cox et al.’s model in predicting discrete risk thresholds under FTI and BTI conditions.

Finally, from a general perspective, it is also worth noting that the term *DV* has more impact on BTI than on FTI (Table 2), and can be therefore interpreted as a descriptor of the water flow. This is in line with the higher impact of such destabilizing hydrodynamic term on the moment balance, due to the lower resistive moment in BTI than in FTI.

### Supporting decision-makers in flood risk assessment and mitigation

Providing a merged BTI-FTI model represents valuable support for practitioners, such as emergency planners and local authorities, in assessing risk levels in pedestrian evacuation using unique outputs while considering: (1) individuals moving towards different directions along defined evacuation routes; (2) uncertainties in (local) trajectories of pedestrians, which can be represented by standard errors in risk map visualization (see Fig. 4cand Fig. 4d).

The results derived from the discretization into three-zone risk levels within a real urban scenario prove coherent with respect to the consolidated deterministic approach^33^. At the same time, they seem to be less conservative than those obtained using the methods proposed by other authors^57,58^. These results hold when adopting $p^*=0.5$ as a reasonable threshold for the three-zone approach. A lower value of $p^*$ would, in turn, yield more conservative risk assessments.

The final “discrete” approach may also be valuable for decision makers in urban contexts, since the logistic models can be used to derive risk assessment maps related to toppling events, thus contributing to basic concepts of flood risk assessment and mitigation^6^. Such maps can support more robust evaluations, including worst-case scenarios and related uncertainties, and help design redundant mitigation strategies for the most vulnerable parts of flood-prone areas. A key innovation is that, for the first time, the mutual direction of pedestrian movement (potentially aligned with evacuation routes) and floodwater spreading is explicitly considered. By integrating the resulting maps with behavioural aspects of flood emergencies, evacuation planning, and mitigation strategies^27–29,31,32^, the three-zone approach can provide practical guidance:

- High-risk zones imply toppling for pedestrians moving in any direction. Here the focus should be on timely evacuation alerts and structural protection or shelter-in-place measures, such as ensuring access to upper floors, raised platforms, or handrails in open spaces, possibly using street furniture.
- Medium-risk zones imply strong instability under BTI but limited instability under FTI. In these cases, evacuation routes become critical–for example, moving upstream when a safe area is reachable–and should be combined with structural measures such as raised platforms or handrails.
- Low-risk zones imply limited instability under BTI. The emphasis may be on support systems for pedestrian movement, again through raised platforms or handrails integrated into street furniture for sustainable use, while applying the same routing strategies suggested for medium-risk zones.

These considerations apply under general floodwater conditions, but local differences can be captured by embedding the proposed equations into microscopic evacuation simulators, where pedestrians are represented as agents moving on a continuous plane or cellular grid^27,29,39,41–43^. Incorporating logistic-based stability rules into such models would allow the evaluation of how directional changes during evacuation may mitigate or exacerbate instability risks^27,29,39^, overcoming the limitations of empirical hazard ratings and traditional multi-zone approaches^40,42,53^. This integration would also enable stability thresholds to be linked with specific pedestrian behaviours and trajectories, and support evacuation path optimization that balances stability risks with evacuation times^43^. While particularly relevant for underground structures, these outcomes can be extended to broader urban safety planning.

### Limitations and future works

Limitations to the approach are essentially linked to the available experimental data used to elaborate the logistic models^36^. For instance, they explore a specific range of $D-V$ pair values that, although representative of the overall conditions covering toppling phenomena, could be expanded in future research attempts. Moreover, additional experimental data referring to other angles between the direction of floodwaters and pedestrian movement could be included to further extend the model’s validity beyond the simplest dichotomous BTI-FTI conditions. Nonetheless, experimental input data still focus on the use of a single physical model at a quasi-natural scale, but could be extended to data at a natural scale, e.g. safely involving volunteers in tests^57^. These tests should be performed by varying $D-V$ pairs along the stability threshold hypothesized in the reference work^36^, rather than just exploring conditions leading to the effective toppling of pedestrians. Eventually, further subject-specific anthropometric information (e.g. weight, height, body mass,etc.) could be easily embedded within the proposed logistic regression framework as additional covariates, whenever available. This would however require additional data from complete experiments that account for relevant human body conditions. Such data could be organized into specific sub-categories for unified FTI and BTI assessment, following the approach of previous works on hazard degree (e.g., stability curves for adults and children^33^) and relative damage functions^47^.

## Supplementary Information

Supplementary Information.

## Data Availability

Code and data are publicly available at https://github.com/minmar94/TopplingRisk
