# Supplementary material for: Probabilistic assessment of human instability in urban areas exposed to flood events
Source: Sci Rep. 2025 Nov 24;15:41505. doi: 10.1038/s41598-025-25267-y (PMC12645039; doi:10.1038/s41598-025-25267-y)
Supplement: Supplementary file 1 — Supplementary Information. [file 41598_2025_25267_MOESM1_ESM.pdf]

Probabilistic assessment of human instability in urban areas exposed to flood events
# – Supplementary Information –

A Additional results

This section includes additional details about the results obtained on the real data application shown in the manuscript.

In particular, Fig A.1 shows the values for water depth (Fig A.1a), water speed (Fig A.1b) and their interaction (Fig A.1c) that have been used to obtain the predicted toppling probabilities. We note that, given the positive association between all the quantities involved in the model, the toppling probabilities mimic the behaviour of the covariates across the study area. In other words, the toppling risk is high/moderate for large water depth and speed values, or both. This is indeed observed in the leftmost part of the study area, which is closer to the flood origin.

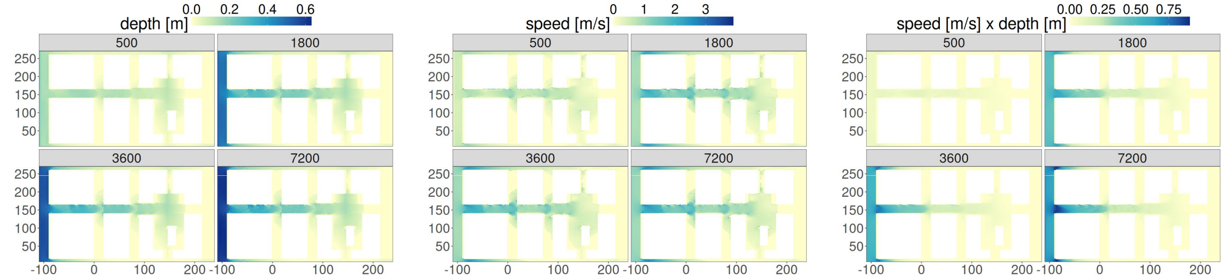

(a) (b) (c)

Figure A.1: Depth (a) speed (b) and their product (c) values on the urban area.

In Fig A.2a and Fig A.2b we show the distribution of the predicted log-odds and related standard errors, respectively for BTI and FTI conditions at different time instants. In all cases, we note that the distribution is at least bimodal, suggesting groups of data with similar toppling risks (and associated uncertainty). In addition, it can be noticed that the distributions become more heterogeneous as time passes, describing a situation where toppling is either certain or impossible.

B Comparison of instability curves

The present section compares the models considered in this work with alternative approaches and the deterministic analysis on the same data by[1] in Figure B.1a and Figure B.1b, respectively. The BTI and FTI laws are represented in red (respectively, solid and dashed lines), while the literature models are shown in black (four-zone approach by Cox et al.[2]), blue (three-zone approach by Martinez-Gomariz et al.[3]) and yellow (two-zone approach by Temez[4]).

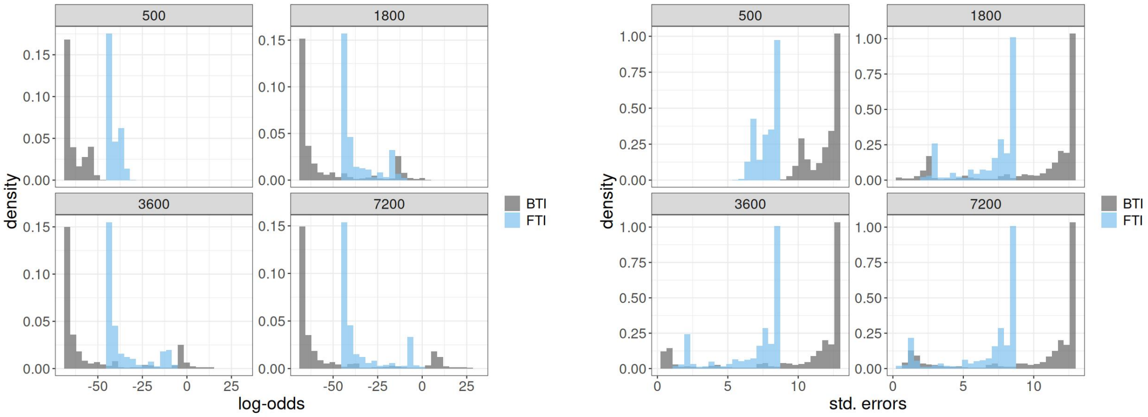

(a) (b)

Figure A.2: Log-odds (a) and associated standard error distribution (b) at different times for BTI and FTI.

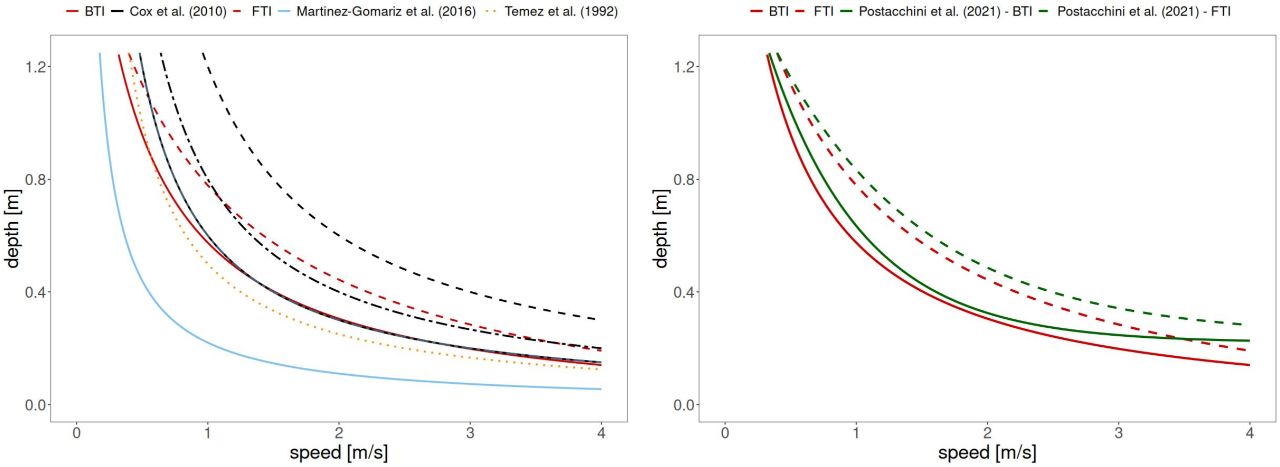

(a) (b)

Figure B.1: Comparison of the risk curves referring to the considered models: (a) present work (red lines, indicating BTI and FTI conditions), Cox et al.[2] (black lines), Martinez-Gomariz et al.[3] (blue lines), Temez[4] (yellow line), (b) Postacchini et al.[1] - BTI (solid blue line) and Postacchini et al.[1] - FTI (dashed green line).

Finally, Table 1 summarizes Fig. 5, by displaying the proportion of pixels assigned to a specific risk area and across methods and times. The first two columns of the table, respectively, report the percentages of pixels with an average toppling probability greater than 0.5 (i.e., using the predicted log-odds of Fig. 3 – panel a) and the percentages of pixels associated with the three risk areas, defined by Fig. 3, panel b.

Table 1: Percentages of urban areas at different risk levels over time and across models.

Time (s)	Our proposal				Martínez-Gomariz et al. (2016)			Temez et al. (1992)			Cox et al. (2010)		
	$p^* > 0.5$	Low	Mod.	High	Low	Mod.	High	Low	Mod.	High	Low	Mod.	High
500	0.0	100.0	0.0	0.0	100.0	0.0	0.0	99.1	–	0.9	100.0	0.0	0.0
1800	0.0	99.5	0.5	0.0	81.4	18.0	0.6	87.5	–	12.5	99.4	0.6	0.0
3600	1.0	96.9	3.1	0.0	80.4	17.2	2.4	77.5	–	22.5	97.6	2.4	0.0
7200	7.9	86.1	13.8	0.1	78.6	7.5	13.9	76.5	–	23.5	86.1	12.8	1.1

C Model validation

To further assess the accuracy of the proposed method in predicting toppling probabilities, we show the ROC curves in Fig. C.1a and Fig, C.1b for BTI and FTI conditions, respectively. Fig. C.1c shows instead the results of the joint predictions. The results obtained highlight a pronounced cusp in all cases, which is typically indicative of the presence of an optimal classification threshold. In other words, such a shape suggests that the predicted probabilities are not continuously distributed but instead cluster around certain values, leading to abrupt changes in the trade-off between true- and false-positive rates (see also Fig. A.2). From a practical standpoint, the cusp highlights a threshold that provides a particularly favorable balance between correctly identifying actual toppling cases and minimizing wrongly classified non-toppling situations as falls.

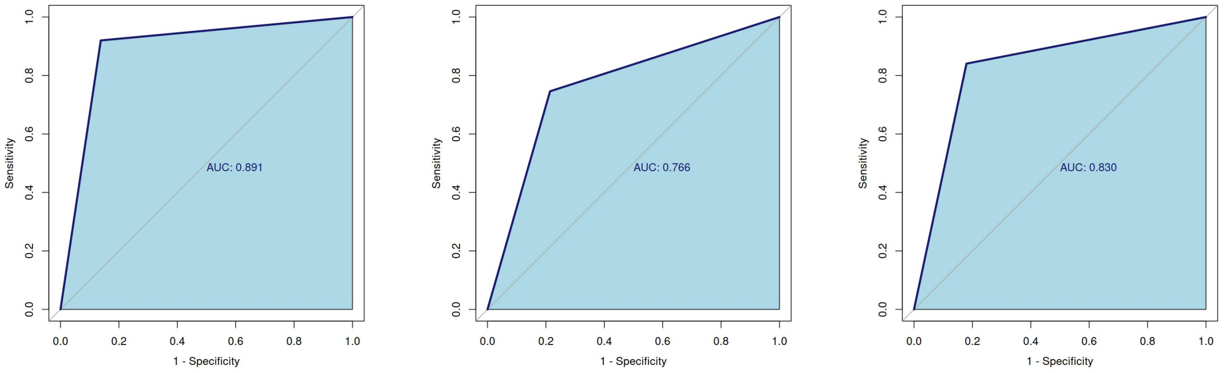

(a) BTI (b) FTI (c) BTI-FTI combination

Figure C.1: ROC curves using predicted probabilities for BTI (panel a), FTI (panel b) and combining them together (panel c).

Finally, we report the calibration plots in Fig. C.2, mimicking the structure of Fig C.1. The calibration plot compares predicted probabilities of toppling events with the corresponding observed frequencies across bins of risk. A well-calibrated model should produce points close to the dashed red line (the first bisector), indicating that predicted risks accurately reflect the empirical probability of toppling. In our case, the plot provides evidence that the model yields probability estimates that are consistent with the observed data, supporting the reliability of the predictions for practical risk assessment in urban flood scenarios.

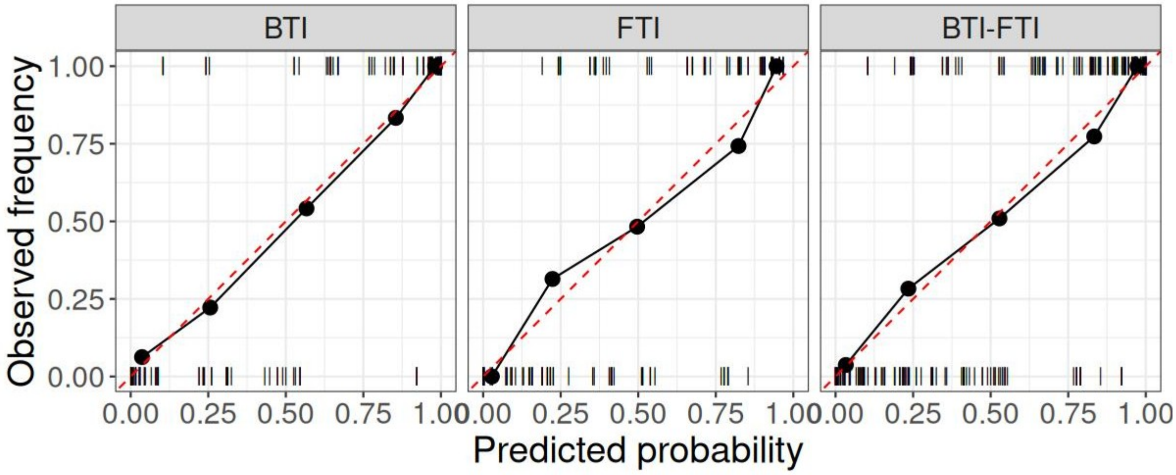

Figure C.2: Calibration plots of the proposed model for BTI and FTI conditions. The dashed red line represents the bisector. The "|" are the observed data points.

Finally, to provide a more robust assessment of predictive performance, we implemented a 10-fold cross-validation for the proposed model. Specifically, we randomly split the sample into 10 groups (each of size 26 or 27), iteratively fitted the model (Model 2 in Table 1) leaving one group out, and used the resulting estimates to predict the outcomes for the left-out group. Prediction accuracy was then computed for each fold, and the average accuracy across folds was 81.2%.

References

[1] Matteo Postacchini, Gabriele Bernardini, Marco D'Orazio, and Enrico Quagliarini. Human stability during floods: Experimental tests on a physical model simulating human body. *Safety science*, 137:105153, 2021.

[2] RJ Cox, TD Shand, and MJ Blacka. Australian rainfall and runoff revision project 10: appropriate safety criteria for people. *Water Res*, 978:085825–9454, 2010.

[3] Eduardo Martínez-Gomariz, Manuel Gómez, and Beniamino Russo. Experimental study of the stability of pedestrians exposed to urban pluvial flooding. *Natural hazards*, 82(2):1259–1278, 2016.

[4] JR Témez. Control del desarrollo urbano en las zonas inundables. *Monografías del Colegio de Ingenieros de Caminos, Canales y Puertos*, 10:105–115, 1992.
